# Validation of the BOADICEA model and a 313-variant polygenic risk score for breast cancer risk prediction in a Dutch prospective cohort

**DOI:** 10.1038/s41436-020-0884-4

**Published:** 2020-07-06

**Authors:** Inge M. M. Lakeman, Mar Rodríguez-Girondo, Andrew Lee, Rikje Ruiter, Bruno H. Stricker, Sara R. A. Wijnant, Maryam Kavousi, Antonis C. Antoniou, Marjanka K. Schmidt, André G. Uitterlinden, Jeroen van Rooij, Peter Devilee

**Affiliations:** 1 Department of Human Genetics, Leiden University Medical Center, Leiden, The Netherlands; 2 Department of Medical Statistics, Leiden University Medical Center, Leiden, The Netherlands; 3 Centre for Cancer Genetic Epidemiology, Department of Public Health and Primary Care, University of Cambridge, Cambridge, United Kingdom; 4 Department of Epidemiology, Erasmus Medical Centre, Rotterdam, The Netherlands; 5 Department of Respiratory Medicine, Ghent University Hospital, Ghent, Belgium; 6 Department of Bioanalysis, Faculty of Pharmaceutical Sciences, Ghent University, Ghent, Belgium; 7 Department of Clinical Genetics, Leiden University Medical Center, Leiden, The Netherlands; 8 Division of Molecular Pathology, the Netherlands Cancer Institute, Amsterdam, The Netherlands; 9 Department of Internal Medicine, Erasmus Medical Centre, Rotterdam, The Netherlands; 10 Department of Pathology, Leiden University Medical Center, Leiden, The Netherlands

**Keywords:** breast cancer, polygenic risk score, prospective cohort, risk assessment

## Abstract

**Purpose:**

We evaluated the performance of the recently extended Breast and Ovarian Analysis of Disease Incidence and Carrier Estimation Algorithm (BOADICEA version 5) in a Dutch prospective cohort, using a polygenic risk score (PRS) based on 313 breast cancer (BC)–associated variants (PRS_313_) and other, nongenetic risk factors.

**Methods:**

Since 1989, 6522 women without BC aged 45 or older of European descent have been included in the Rotterdam Study. The PRS_313_ was calculated per 1 SD in controls from the Breast Cancer Association Consortium (BCAC). Cox regression analysis was performed to estimate the association between the PRS_313_ and incident BC risk. Cumulative 10-year risks were calculated with BOADICEA including different sets of variables (age, risk factors and PRS_313_). C-statistics were used to evaluate discriminative ability.

**Results:**

In total, 320 women developed BC. The PRS_313_ was significantly associated with BC (hazard ratio [HR] per SD of 1.56, 95% confidence interval [CI] [1.40–1.73]). Using 10-year risk estimates including age and the PRS_313_, other risk factors improved the discriminatory ability of the BOADICEA model marginally, from a C-statistic of 0.636 to 0.653.

**Conclusions:**

The effect size of the PRS_313_ is highly reproducible in the Dutch population. Our results validate the BOADICEA v5 model for BC risk assessment in the Dutch general population.

## INTRODUCTION

Breast cancer is the most common cancer among women in Europe.^1^ In the Netherlands, the average lifetime risk for developing invasive breast cancer is 13.6% for each woman, with the incidence peaking between 60 and 70 years of age.^2^ Mammographic screening has decreased breast cancer mortality at the cost of detecting more disease that otherwise would not have become clinically apparent.^3,4^ Based on the UK guidelines, for every 10,000 women invited for screening at age 50 for the following 20 years, 43 deaths would be prevented, while 129 breast cancers would be overdiagnosed.^5^ Furthermore, breast cancer screening inevitably yields false positives, which can lead to anxiety.^6^ Improvement of this benefit-to-harm ratio could be achieved by targeting women who benefit the most from screening, in particular those in the highest risk categories, while reducing screening for those in the lowest risk categories, potentially reducing overdiagnosis and costs while maintaining a reduced breast cancer death rate and improved quality of life.^7^

Many risk prediction algorithms have been developed to quantify the combined effect of various risk factors to predict the risk of developing breast cancer.^8,9^ The recently extended Breast and Ovarian analysis of Disease Incidence and Carrier Estimation Algorithm (BOADICEA) calculates cumulative risk of developing breast cancer based on family history, mammographic density, and several lifestyle/hormonal and genetic risk factors.^10^ BOADICEA includes the rare high to moderate risk pathogenic variants in breast cancer genes *BRCA1*, *BRCA2*, *PALB2*, *CHEK2*, and *ATM*, and a polygenic risk score (PRS) based on 313 breast cancer–associated variants (PRS_313_). In ten prospective studies, this PRS showed an association with breast cancer with an odds ratio (OR) of 1.61 per standard deviation (SD) of the PRS distribution,^11^ and an area under receiver–operator curve of 0.630. It has been shown that the greatest breast cancer risk stratification in the general population and in women with a family history of breast cancer can be obtained by using the combined effects of the PRS and lifestyle/hormonal risk factors in the BOADICEA model.^10^

Currently, breast cancer screening in the Dutch population is age-based.^12^ Women start at age 50 years with biannual mammograms until the age of 75. Before considering risk-stratified approaches based on BOADICEA, it is important to assess its clinical validity in the Dutch population. In this study we validated the association between the PRS_313_ and breast cancer in a Dutch prospective cohort, its effect on predicting in situ breast cancer, and explore the discriminative ability of an individualized 10-year breast cancer risk score based on the PRS_313_ and several known risk factors using the BOADICEA version 5 model. We also assessed how a risk-based approach of population-based screening could have impacted breast cancer detection rates in our study cohort.

## MATERIALS AND METHODS

### Study cohort

The Rotterdam Study (RS) is a prospective population-based cohort study of elderly Dutch individuals living in the Ommoord district of Rotterdam in the Netherlands.^13^ Briefly, in the year 1989, individuals aged 55 or older were recruited into the RS-I cohort, which was extended in 2000 under similar criteria (RS-II cohort) and in 2006 by the inclusion of individuals with an age between 45 and 55 (RS-III cohort). The overall response rate was 72%. In 2008 the Rotterdam Study comprised 14,926 subjects aged 45 years or older, including 8823 women. For our study, we included all 6670 women for whom genotype data were available. Genotyping was not performed for the excluded 2153 women because of a low-quality DNA sample or because they declined blood donation for DNA at study entry.

### Ethics statement

The Rotterdam Study has been approved by the Medical Ethics Committee of the Erasmus Medical Center and by the Dutch Ministry of Health, Welfare, and Sports. All participants provided written informed consent to participate in the study and to have their medical information obtained from treating physicians.

### Phenotype data

Diagnoses of cancer were collected for all individuals up to January 2014 and were based on medical records of general practitioners (including hospital discharge letters) and through linkage with Dutch Hospital Data, Netherlands Comprehensive Cancer Organisation, and histology and cytopathology registries in the region.^13^ In total, 468 women had a breast cancer (invasive or in situ) diagnosis of whom 148 had been diagnosed prior to entry into the Rotterdam Study, and were excluded from further analyses. All participants were interviewed at home at inclusion, underwent extensive examinations every ~5 years in the Rotterdam Study research facility, and received follow-up questionnaires (Fig. S1), as described elsewhere.^13^ Basic characteristics such as date of birth, vital status, and age at inclusion were known for all participants. For most participants, information on breast cancer risk factors was available (Table S1, total cohort), but family history of breast cancer and mammographic density were lacking. For the analyses, we used only information from the first questionnaire (Fig. S1: RS-I-1, RS-II-1, RS-III-1) at the time of inclusion in the Rotterdam Study for variables that could vary over time, e.g. weight and alcohol use. Age at menopause was only included if menopause occurred before enrollment into the Rotterdam Study (Table S1, subcohort).

### Genotype data

Genotyping was performed with the Illumina 550 K (RS-I and RS-II cohorts) and 610 K (RS-III cohort) arrays.^13^ Standard quality control was completed, including selection on European ancestry, and imputation was performed using the Haplotype Reference Consortium (HRC) 1.1 and 1000 G phase 3 reference panels.^14,15^ Of the 313 variants used to calculate the PRS, 28 were directly genotyped by the arrays. Two variants were imputed with a quality below 0.3 and the remaining 283 variants were imputed with an average imputation quality of 0.95 (Table S2).

### Polygenic risk score calculation

The following formula was used to calculate the PRS based on 313 variants:

$$PRS_j = \mathop {\sum}\limits_{i = 1}^{313} {n_{ij}\,{\mathrm{ln}}\left( {OR_i} \right)}$$

where *n*_*ij*_ is the number of risk alleles (0, 1, or 2) for variant *i* carried by individual *j* and *OR*_*i*_ is the per-allele OR for breast cancer associated with variant *i*. The ORs were obtained from the Breast Cancer Association Consortium (BCAC) study^11^ (Table S2). As the estrogen receptor (ER) status of the breast tumors was not available, only the overall breast cancer PRS was calculated. The PRS_313_ was standardized to the mean in all included women from the Rotterdam Study who did not develop incident breast cancer. To allow for direct comparison of PRS performance between both studies, the SD of the population controls included in the validation set from the BCAC study^11^ was used, which was 0.609. For the calculations with BOADICEA version 5, the PRS_313_ was standardized to the mean and SD from the population controls included in the total data set from the BCAC study,^11^ which were −0.424 and 0.603 respectively.

### Cumulative risk score calculation

Cumulative 10-year breast cancer risks were calculated with BOADICEA version 5,^10^ starting at the age of inclusion in the Rotterdam Study, and using the birth cohort incidence rates in combination with four different sets of variables, i.e., (1) age, (2) age and PRS_313_, (3) age and risk factors, and (4) age, PRS_313_, and risk factors. Risk factors included are age at menarche, age at menopause, number of children, age at first live birth, use of oral contraception, use of hormone replacement therapy, body mass index (BMI), height, and alcohol use. For the variables that could vary over time, we used fixed variables. As BOADICEA ignores any risk factors for which the value is missing,^10^ no imputation was performed, and missing variables were kept missing.

Because BOADICEA calculates cumulative breast cancer risks up to age 80, 10-year breast cancer risks were only calculated for 4377 women with an age of inclusion up to 70 years. Women were considered affected if they developed breast cancer (invasive or in situ) within 10 years after inclusion in the Rotterdam Study.

### Statistical analyses

Cumulative incidences were calculated using the Kaplan–Meier method.

#### Association analyses

To estimate the association between the PRS_313_ and breast cancer risk in the Rotterdam Study cohort, Cox regression analyses were performed. Relatedness among individuals of the same family was accounted for by correcting standard errors using a sandwich estimator. All models were adjusted by the age at inclusion in the Rotterdam Study. Incident breast cancer, in situ or invasive, was the event of interest. The time at risk was defined as the time elapsed between the inclusion date and the date of occurrence of the event of interest or right censoring. Right censoring could be due to (1) end of follow-up in January 2014 or (2) death. The proportional hazard assumption for the model was tested. Sensitivity analyses were performed (1) for invasive breast cancer only by censoring the in situ breast cancer cases, (2) for in situ breast cancer only by censoring the invasive breast cancer cases, (3) by censoring at the age of diagnosis of another type of cancer, and (4) by stratifying on Rotterdam Study cohort. To define the association between the PRS_313_ and other tumors than breast cancer, similar Cox regression analysis was performed by censoring the breast cancer cases if they did not develop another tumor before the breast cancer diagnosis.

To investigate if the linearity assumption for the effect of PRS_313_ holds, we ran the model considering the categorical covariate given by the percentile groups of the PRS_313_ (0–10%, 10–20%, 20–40%, reference 40–60%, 60–80%, 80–90%, 90–100%) based on the distribution in the unaffected women in this cohort. The discrimination ability of the PRS_313_ in our sample was evaluated using the C-statistic,^16^ by groups based on quantiles of the age of inclusion in the Rotterdam Study (i.e. age <60, 60–70, and ≥70 years). Differences in the C-statistics were tested by computing bootstrap CIs for the differences among groups.

#### Age-varying effect

The possible time-varying association of the PRS_313_ with breast cancer was investigated using age as time scale and considering three age-dependent coefficients in the Cox model, corresponding to three different age intervals: (1) younger than 50 years, (2) between 50 and 75 years old, and (3) above 75 years old. These cut-offs were chosen based on their clinical relevance since women between 50 and 75 years are eligible for population screening according to the Dutch guideline.^12^

#### Clinical validity of BOADICEA v5

To validate the BOADICEA 10-year cumulative risk scores, model calibration and discrimination ability in our sample were assessed. Calibration was investigated by comparing overall observed versus expected cumulative risks and by visually inspecting the calibration plots based on risk deciles. Because of the presence of right censoring, empirical risks at 10 years were estimated using the Kaplan–Meier method. As in the association analyses, discrimination was evaluated using C-statistics.

Statistical significance was defined as a two-sided *p* value of <0.05. All analyses were performed with R version 3.5.3.^17^

## RESULTS

We included 6522 women in the main analyses with an average age at study entry of 66 years. Of these, 320 developed either invasive or in situ breast cancer during follow-up and 744 developed another type of tumor; the overlap between these two groups was 16, all of whom developed another type of tumor first (Table S3). The median follow-up calculated with the reverse Kaplan–Meier method was 12.40 years, with a minimum and maximum follow-up of 0.03 and 24.43 years. Cohort characteristics are shown in Table S1. The average PRS_313_ in groups of affected (i.e. invasive, in situ, and a second breast tumor) and unaffected women (including women who developed another tumor than breast cancer) are shown in Fig. S2 and Table S4.

### Breast cancer cumulative incidence

The cumulative incidence of breast cancer in the total cohort was on average 4.2%, 95% CI [3.7%–4.8%] and 7.3%, 95% CI [6.4%–8.2%] 10 and 20 years after inclusion respectively. Stratified by quintiles of the PRS_313_, after 20 years of follow-up, the incidence in the highest quintile was 10.8%, 95% CI [8.5%–13.1%] and 4.4%, 95% CI [2.8%–6.0%] for the lowest quintile (Fig. S3).

### Association analyses

A significant association was found between the PRS_313_ and incident breast cancer with an HR per SD of 1.56, 95% CI [1.40–1.74], *p* = 2.47 × 10^−15^ (Table 1). There was no evidence of violation of the proportional hazard assumption (*p* value = 0.716), indicating that the HR remained constant over time. The discriminative ability of the PRS_313_, as measured by the C-statistic, was 0.632, 95% CI [0.58–0.69], 0.673, 95% CI [0.61–0.73], and 0.562, 95% CI [0.48–0.62] for women included before age 60, between age 60 and 70, and above age 70 respectively (Table 1).

**Table 1:** Results of the association analyses between breast cancer and the PRS_313_.

	*n* Included	*n* Events	HR	95% CI	*p* value	C-statistic^c^	95% CI
Main analyses	6522	320	1.56	1.40–1.74	2.47×10^−15^		
Age category for discriminative ability of the PRS							
<60	2175	104				0.632	0.58–0.69
60–70	2174	128				0.673	0.61–0.73
≥70	2173	88				0.562	0.48–0.62
Sensitivity analyses							
Invasive BC only	6522	290^a^	1.57	1.40–1.77	1.34 × 10^−14^		
In situ BC only	6522	34	1.43	1.01–2.01	0.042		
Censored at other tumor	6402^b^	298	1.54	1.37–1.73	1.88 × 10^−13^		
Stratified by RS cohort	6522	320	1.56	1.40–1.75	1.92 × 10^−15^		
Percentage of the PRS							
0–10%	637	17	0.59	0.34–1.01	0.053		
10–20%	636	16	0.58	0.33–1.01	0.053		
20–40%	1283	42	0.73	0.49–1.09	0.120		
40–60%	1298	57	1.00	Reference	Reference		
60–80%	1325	85	1.49	1.07–2.09	0.019		
80–90%	656	36	1.28	0.84–1.94	0.251		
90–100%	687	67	2.37	1.66–3.37	1.73 × 10^−06^		
Age category for time-varying analyses							
<50	224	2	2.74	1.72–4.37	2.23 × 10^−05^		
50–75	5104	197	1.74	1.52–2.00	2.21 × 10^−15^		
>75	4032	121	1.29	1.08–1.55	0.005		

*BC* breast cancer, *CI* confidence interval, *HR* hazard ratio, *PRS* polygenic risk score, *RS* Rotterdam Study.

^a^4 women developed an invasive breast tumor after development of an in situ breast tumor.

^b^120 women were excluded from analyses because they developed another tumor before inclusion in the Rotterdam Study.

^c^The corresponding differences in C-statistic were for women with inclusion age 60–70 versus age <60: 0.041, 95% CI [−0.05–0.12]; for women with inclusion age 60–70 versus age ≥70: 0.111, 95% CI [0.02–0.21]; for women with inclusion age <60 versus age ≥70: 0.070, 95% CI [−0.01–0.18].

Sensitivity analyses for (1) invasive breast cancer only, (2) censoring at another tumor if applicable, or (3) stratifying by the Rotterdam Study subcohort all showed similar results (Table 1). Notably, in situ breast cancer also showed a statistically significant association with the PRS_313_, HR per SD = 1.43, 95% CI [1.01–2.01], *p* = 0.042.

**Fig. 1:**
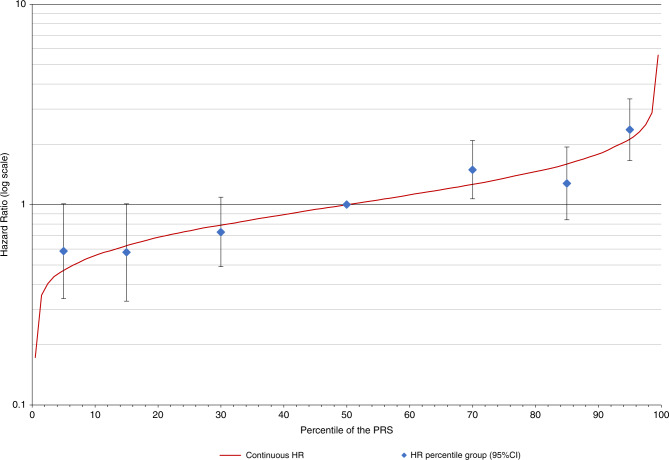
Association with the PRS_313_ and breast cancer risk. Plot of the HR for the association between the PRS_313_ and breast cancer risk based on PRS_313_ percentiles. The PRS_313_ percentile groups are 0–10%, 10–20%, 20–40%, 40–60% (reference), 60–80%, 80–90%, 90–100% based on the distribution in unaffected women. The numbers and corresponding effect sizes are shown in Table 1. The solid line represents the continuous distribution based on the per SD effect size of the PRS_313_. *CI* confidence interval, *HR* hazard ratio, *PRS* polygenic risk score.

Association analyses for breast cancer and percentiles of the PRS_313_ showed that the HR estimates were in line with the HR predicted when a continuous PRS_313_ is assumed, under a log-linear model (Fig. 1, Table 1).

During follow-up, 744 women developed a tumor other than breast cancer without evidence for association with the PRS_313_ (HR per SD = 1.05, 95% CI [0.98–1.12], *p* value = 0.195).

### Age-varying effect

Extension of the Cox model allowing for age-dependent regression coefficients showed that the performance of the PRS_313_ decreased with increasing inclusion age, with the HRs per SD declining from 2.74, 95% CI [1.72–4.37] for women included before age 50, to 1.74, 95% CI [1.52–2.00] for women included between 50 and 75 (*p*_diff_ = 0.066). The HR for women included after age 75 was 1.29, 95% CI [1.08–1.55], and the *p* value of the difference with respect to the youngest group was 0.003 (Table 1).

### Clinical validity of BOADICEA V5

For these analyses, we selected 4377 women with an age of inclusion under 70 years. Of these, 163 developed breast cancer within 10 years after inclusion (142 invasive). The median follow-up in this subcohort was 10 years (range 0.03–10 years), and the cumulative incidence of breast cancer was 4.4% (95% CI [3.7–5.1%]). The distributions of 10-year cumulative risk scores under different models are shown in Figs. S4 and S5. Irrespective of the variables included, BOADICEA underestimated the observed risk of 4.4% (Table 2). Accordingly, while using age and PRS_313_ seems to result in the best calibration (Fig. S4C), it underestimated the observed risks in the higher risk categories. The highest discriminative ability was found for the model with age, PRS_313_ and all available risk factors (0.653, 95% CI [0.60–0.70]), henceforth the “full” model. The PRS_313_ was the strongest factor contributing to discrimination, relative to age and other risk factors (Table 2).

**Table 2:** Range and discriminative ability of the cumulative 10-year breast cancer risk scores calculated with BOADICEA.

Variables included	Mean % (range)		C-statistic	95% CI
	Unaffected women	BC cases^a^		
Age	3.0 (2.2–3.6)	2.9 (2.2–3.6)	0.531	0.50–0.58
Age, risk factors	2.5 (1.0–5.9)	2.6 (1.4–4.3)	0.558	0.52–0.60
Age, PRS_313_	3.1 (0.6–11.9)	3.8 (1.2–8.3)	0.636	0.59–0.68
Age, risk factors, PRS_313_	2.6 (0.4–11.4)	3.3 (0.9–10.5)	0.653	0.60–0.70

*BC* breast cancer, *BOADICEA* Breast and Ovarian Analysis of Disease Incidence and Carrier Estimation Algorithm, *CI* confidence interval, *PRS* polygenic risk score.

^a^Women who developed BC within 10 years of follow-up.

**Fig. 2:**
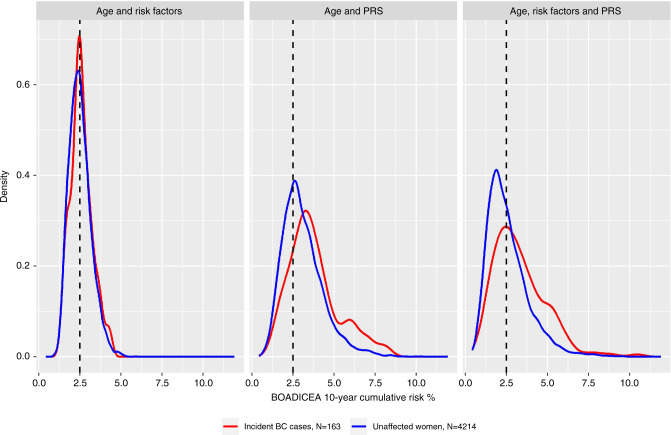
Cumulative 10-year breast cancer risk distribution predicted by BOADICEA. Density plots of the cumulative 10-year risk calculated by BOADICEA for unaffected women and incident breast cancer cases. Including age and risk factors (left), including age and the PRS_313_ (middle), and the full model including age, risk factors and the PRS_313_. The dashed line shows the threshold of a 10-year risk of 2.5%. *BOADICEA* Breast and Ovarian Analysis of Disease Incidence and Carrier Estimation Algorithm, *PRS* polygenic risk score.

Using the full model and a threshold of 2.5% 10-year breast cancer risk, which approximates the risk of women entering the age-based population screening program in the Netherlands, 101 cases (62% of incident cases) occurred in a screening group of 1956 women (45% of total) and 62 breast cancers occurred in 2421 women who would not be screened (Fig. 2; Table 3). Using the PRS_313_ and age only, 130 cases (80% of incident cases) occurred in a screening group of 2863 women (65% of total); in 1481 women who would not be screened, 33 breast cancers occurred. In Fig. S6 the percentages of incident breast cancer cases and unaffected women are shown for different category thresholds. For both models, the invasive cancers in the group selected for screening were more likely to be of lower grade compared with the cancers in the nonscreened group (Table 3). The reverse effect was found for in situ cancers.

**Table 3:** Numbers and percentages of women per 10-year risk category.

		10-year risk category based on BOADICEA			
	Total	Including age and PRS		Including age, risk factors, and PRS	
		<2.5%	>2.5%	<2.5%	>2.5%
Unaffected women					
All	4214	1481 (35%)	2733 (65%)	2359 (56%)	1855 (44%)
Incident BC cases					
All	163	33 (20%)	130 (80%)	62 (38%)	101 (62%)
Invasive BC					
All	142	30 (21%)	112 (79%)	52 (37%)	90 (63%)
Grade 1	19	2 (11%)	17 (89%)	3 (16%)	16 (84%)
Grade 2	38	7 (18%)	31 (82%)	12 (32%)	26 (68%)
Grade 3	43	13 (30%)	30 (70%)	21 (49%)	22 (51%)
Unknown	42	8 (19%)	34 (81%)	16 (38%)	26 (62%)
In situ BC					
All	21	3 (14%)	18 (86%)	10 (48%)	11 (52%)
Grade 1	3	2 (67%)	1 (33%)	2 (67%)	1 (33%)
Grade 2	3	1 (33%)	2 (67%)	2 (67%)	1 (33%)
Grade 3	13	0 (0%)	13 (100%)	5 (38%)	8 (62%)
Unknown	2	0 (0%)	2 (100%)	1 (50%)	1 (50%)

*BC* breast cancer, *BOADICEA* Breast and Ovarian Analysis of Disease Incidence and Carrier Estimation Algorithm, *PRS* polygenic risk score.

## DISCUSSION

Many risk factors for breast cancer, both genetic and nongenetic, have been identified in the past decades.^18,19^ Increasingly, these are being integrated into computational models that allow personalized breast cancer risk assessment, which has potential application beyond current practice of genetic testing in family cancer clinics.^8,9,20^ The BOADICEA algorithm is among the most comprehensive risk models presently available for breast cancer risk assessment.^10^ Here, we validated the most recent version of this model in a large prospective population-based Dutch cohort of women above 45 years, which has not been part of the previously published BCAC study.^11^ Unsurprisingly, the best discrimination was achieved after inclusion of all available risk factors, with the largest contribution deriving from the PRS_313_. The PRS_313_ was significantly associated with breast cancer, with a similar effect size as in other prospective series of different geographic origin,^11^ demonstrating its robustness and potential application to the Dutch population.

The PRS_313_ improved the discriminatory ability from 0.531 to 0.636, compared with a model using age only, which could only be marginally improved further (to 0.653) by adding lifestyle, reproductive factors, and anthropometric data. This is in line with previous research, showing that the variance explained by the risk factors is modest compared with the PRS_313_ risk stratification.^10,21^ Results of the calibration showed that BOADICEA underestimated the observed risks, especially in the higher categories of risk. One possible explanation is that BOADICEA v5 uses the population breast cancer incidences of the United Kingdom as baseline risk, which are slightly lower than those in the Netherlands.^1^ But more importantly, data on family history, mammographic density, and rare high-risk variants in *BRCA1* and *BRCA2* were lacking in our cohort. In another prospective validation study of a previous version of BOADICEA in two cohorts of women from Australia, Canada, and the United States, information on family history and *BRCA1/2* carrier status, but not the PRS_313_, was available, and here, BOADICEA overestimated 10-year cumulative risks in the highest risk quantile.^9^ Possibly, the missing data on family history and *BRCA1/2* status in the Rotterdam Study were in fact more prevalent than modeled by BOADICEA. Our calibration results indicate that for proper use in the general population, information on family history may be important.

We illustrated the potential impact of the model in detecting breast cancer in a population screening setting in which women would participate based on their individual risk. In this illustration, the PRS_313_ alone would have detected more cases than the full BOADICEA model, but would also have identified a larger screening group. Apparently, women in the Rotterdam Study have on average fewer nongenetic risk factors compared with the total population, which on average slightly modifies their risk in a downward direction. The PROCAS study used the Tyrer–Cuzick model with mammographic density and risk factors, combined with a PRS based on 18 single-nucleotide polymorphisms (SNPs);^22^ they found 82% of the cases to occur in 68% of women with a 10-year breast cancer risk above 2%, i.e., very similar to what we found with the PRS_313_ alone.

Remarkably, we found the proportion of low grade invasive tumors to be higher in those with a 10-year risk >2.5%, compared with those with lower risks. Screen-detected invasive cancers are more likely of lower grade and stage.^23^ Our cohort data did not include information on whether incident breast cancers were screen-detected or not, hence we cannot exclude that high-risk women disproportionally self-selected for mammographic screening, which could explain this bias. In contrast, for the in situ carcinomas, more high grade tumors were found in the >2.5% 10-year risk group compared with those with lower risks. Histological grade of ductal carcinoma in situ (DCIS) has been suggested to be one of six factors associated with subsequent development of invasive disease,^24^ albeit not very strongly so. It remains possible that the PRS_313_ is more strongly associated with low grade invasive breast cancer than with higher grades, as observed for some individual variants,^25,26^ and inversely so for DCIS. It will be important to replicate this in larger studies to inform the evaluation of the cost-effectiveness of a risk-based versus age-based entry of the population screening.^7^

Although PRS development studies have included only invasive breast cancer,^11,27^ in our cohort the PRS_313_ is associated with in situ breast cancer as well, with a nonsignificantly lower effect size than for invasive breast cancer. This corresponds well with a previously reported association of an 18-SNP-based PRS^22^ and with previous results showing that the association of 51 of the 76 investigated breast cancer loci with DCIS is in the same direction as for invasive breast cancer.^28^ Although BOADICEA is presented as a model that predicts invasive breast cancer,^10^ these results suggest it might also predict in situ breast cancer. Larger studies are needed to confirm this and provide more accurate risk estimates, specifically in the setting of population screening programs.

As in previous studies,^11,27^ we found that the effect size of the PRS for breast cancer declined with increasing age. While this is not yet modeled in BOADICEA, this could be important to consider for women under the age of 50 who are at this moment not eligible for population breast cancer screening in the Netherlands, because our results suggest that using the overall HR would be underestimating risk in this age group.

In the Rotterdam Study, malignancies other than breast cancer are also recorded. We found no evidence for association of the PRS_313_ with these cancers, suggesting it specifically predicts breast cancer. Another prospective study also reported no association between other types of cancer and a sum of breast cancer risk alleles at 72 loci.^29^ Because we only analyzed all other tumors combined, we cannot exclude that the PRS_313_ has an association with one specific type of other cancer.

A strength of our study is the prospective population-based study design, including all women in a specified locale near Rotterdam. Because of the high response rate (>70%) it is a good representation of the Dutch population in that age category.^13^ Furthermore, for a large group of women, there is extensive follow-up of up to 25 years.

Besides that information on mammographic density and family history was lacking, another limitation of our study is the unknown ER status of the breast tumors, precluding the analysis of ER-positive and ER-negative disease separately. Furthermore, to evaluate the introduction of risk-based entry into population screening, establishing the detection rate of breast cancers below the age of 50 would have been relevant, which was not possible in our older cohort of women. Finally, we excluded nearly 25% of all women in the Rotterdam Study because no genotyping data were available. Declining blood donation for DNA extraction did not lead to differences in the basic characteristics between the genotyped and nongenotyped groups. Therefore, if a selection bias was present, we believe this bias would be small.

In summary, the PRS_313_ replicates robustly in the Dutch population and the discriminative power of the BOADICEA model seems appropriate for implementation into breast cancer prevention programs, such as those currently ongoing in cancer family clinics in many countries worldwide. However, application to the general population would require recalibration of BOADICEA to address underestimation in the higher risk categories. Although the Rotterdam Study design precluded analysis of breast cancer–specific mortality, our evaluation of clinical validity provides first insights into how a risk-based entry could impact the efficacy of the breast cancer population screening program in the Netherlands.

## Supplementary information

Supplementary Information
