# Supplementary material for: Validation of the BOADICEA model and a 313-variant polygenic risk score for breast cancer risk prediction in a Dutch prospective cohort
Source: Genet Med. 2020 Jul 6;22(11):1803–11. doi: 10.1038/s41436-020-0884-4 (PMC7605432; doi:10.1038/s41436-020-0884-4)
Supplement: Supplementary file 1 — Supplementary Information [file 41436_2020_884_MOESM1_ESM.pdf]

Supplementary information

Lakeman et al. Validation of the BOADICEA model and a 313-variant polygenic risk score for breast cancer risk prediction in a Dutch prospective cohort

Supplementary figures

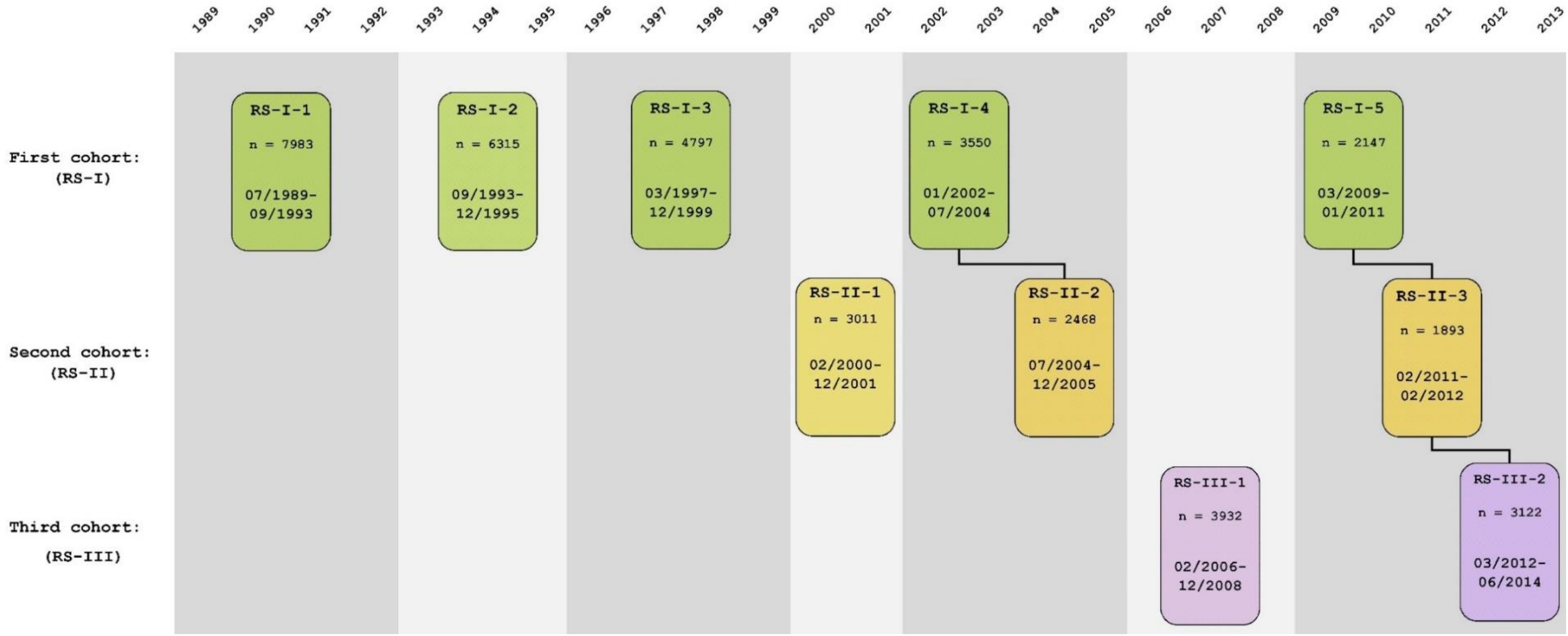

Figure S1: Time points of the first and follow up questionnaires for the Rotterdam Study cohorts

Diagram of the included individuals and contact moments for the Rotterdam Study. Green boxes: RS-I cohort, yellow boxes: RS-II cohort; purple boxes: RS-III cohort. A more elaborate figure with information about the follow up after 2013 is published by Ikram et al.[1].

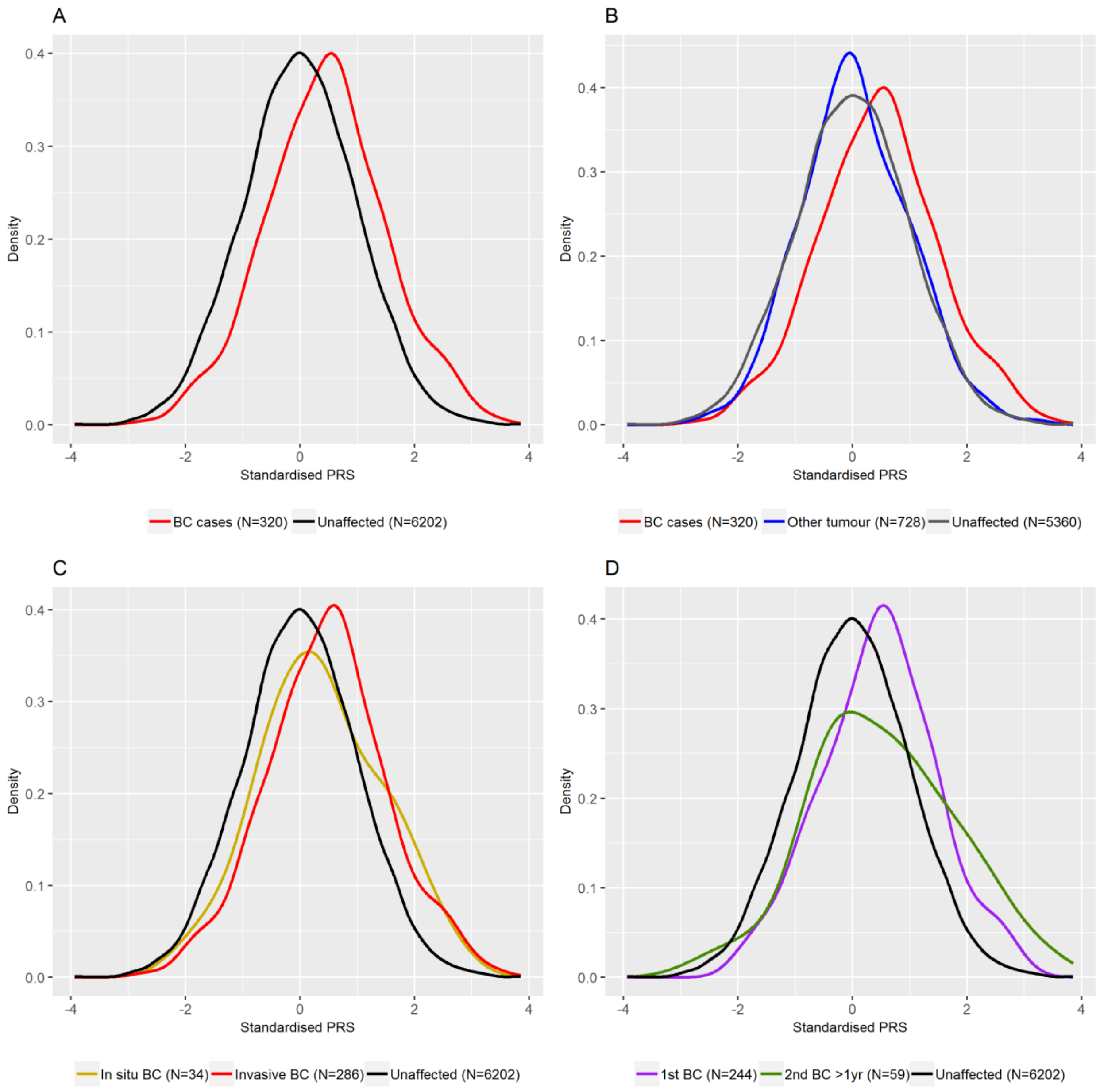

Figure S2: Distribution curves of the PRS_{313} in the Rotterdam Study cohort

Abbreviations: BC, Breast Cancer; PRS, Polygenic Risk Score

The standardised PRS_{313} was plotted against the density for different groups in the Rotterdam Study. (A) incident BC cases and unaffected women; (B) incident BC cases, unaffected women who developed another type of tumour and unaffected women who did not develop another type of tumour. Women who developed another type of tumour before inclusion in the Rotterdam Study were excluded (N=114); (C) invasive incident BC cases, *in situ* incident BC cases and unaffected women; (D) Incident BC cases who developed one breast tumour, incident BC cases who developed a second primary breast tumour after one year and unaffected women. Women who developed a second primary breast tumour within one year were excluded (N=17). Unaffected women include all those that did not develop BC.

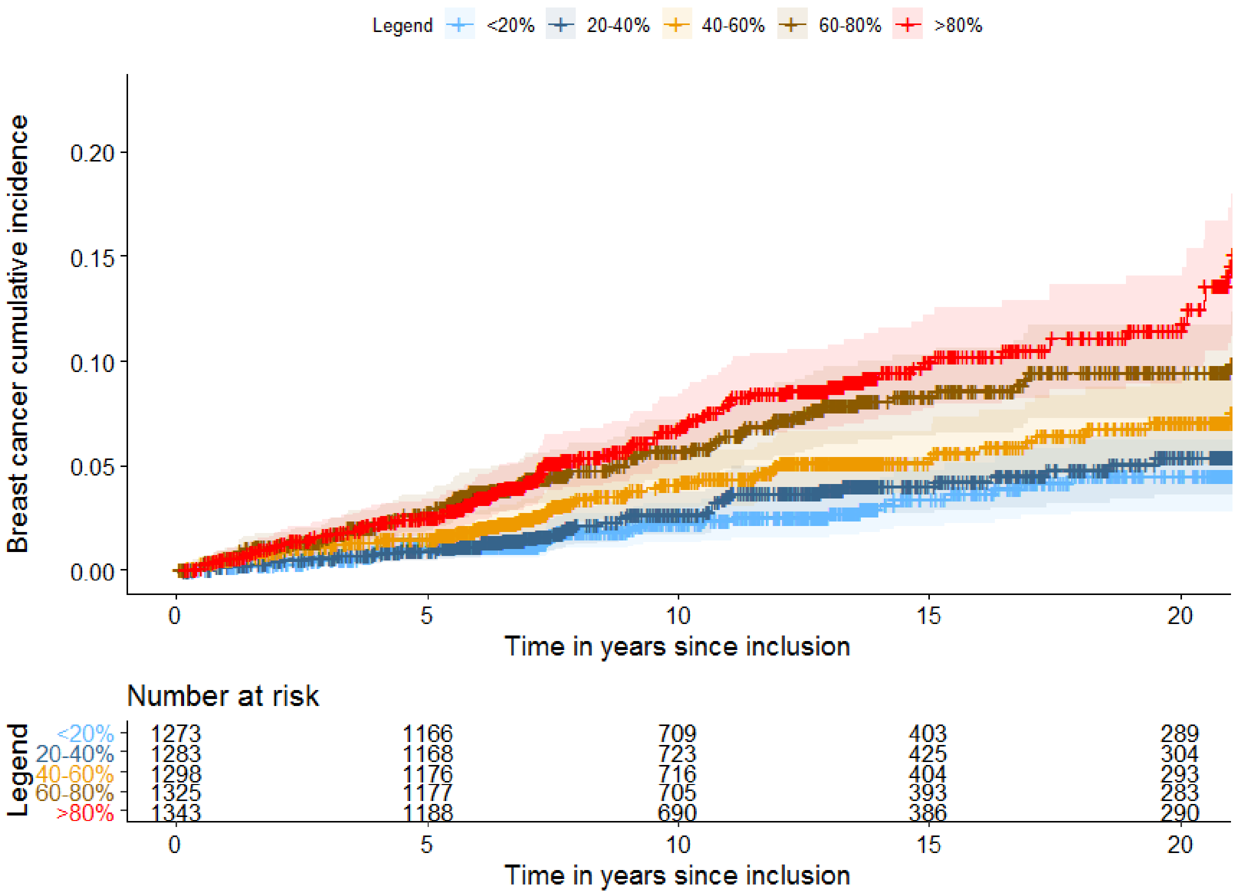

Figure S3: Cumulative breast cancer incidence in the Rotterdam study stratified on PRS_{313} quintiles

Abbreviations: PRS, Polygenic Risk Score.

Kaplan Meier plot for the cumulative breast cancer incidence since the time of inclusion in the Rotterdam Study. The cohort is stratified in quintiles of the PRS_{313}, based on the distribution of unaffected women in the cohort.

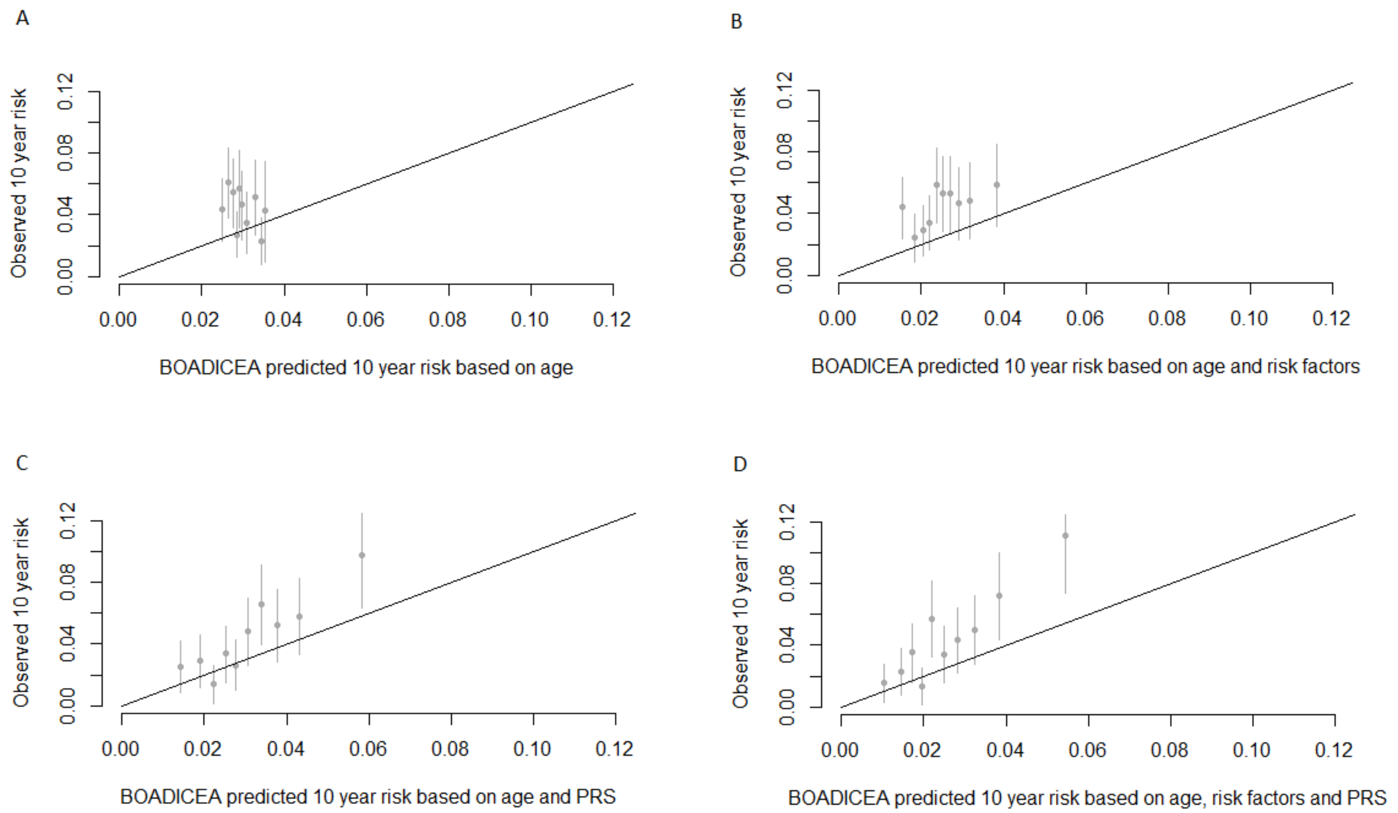

Figure S4: Calibration plots of the predicted 10-year risk based on BOADICEA and the observed risk in the Rotterdam Study cohort

Abbreviations: BOADICEA, Breast and Ovarian Analysis of Disease Incidence and Carrier Estimation Algorithm; PRS, Polygenic Risk Score. 10-year cumulative BC risks were calculated for all women included in the Rotterdam Study before the age of 70 years, using BOADICEA v5. The difference between the observed and predicted risk is shown per decile of the predicted risk, including 95% confidence intervals, for different sets of included variables. Using age only (A), age and risk factors (B), age and the PRS (C) and age, risk factors, and the PRS (D).

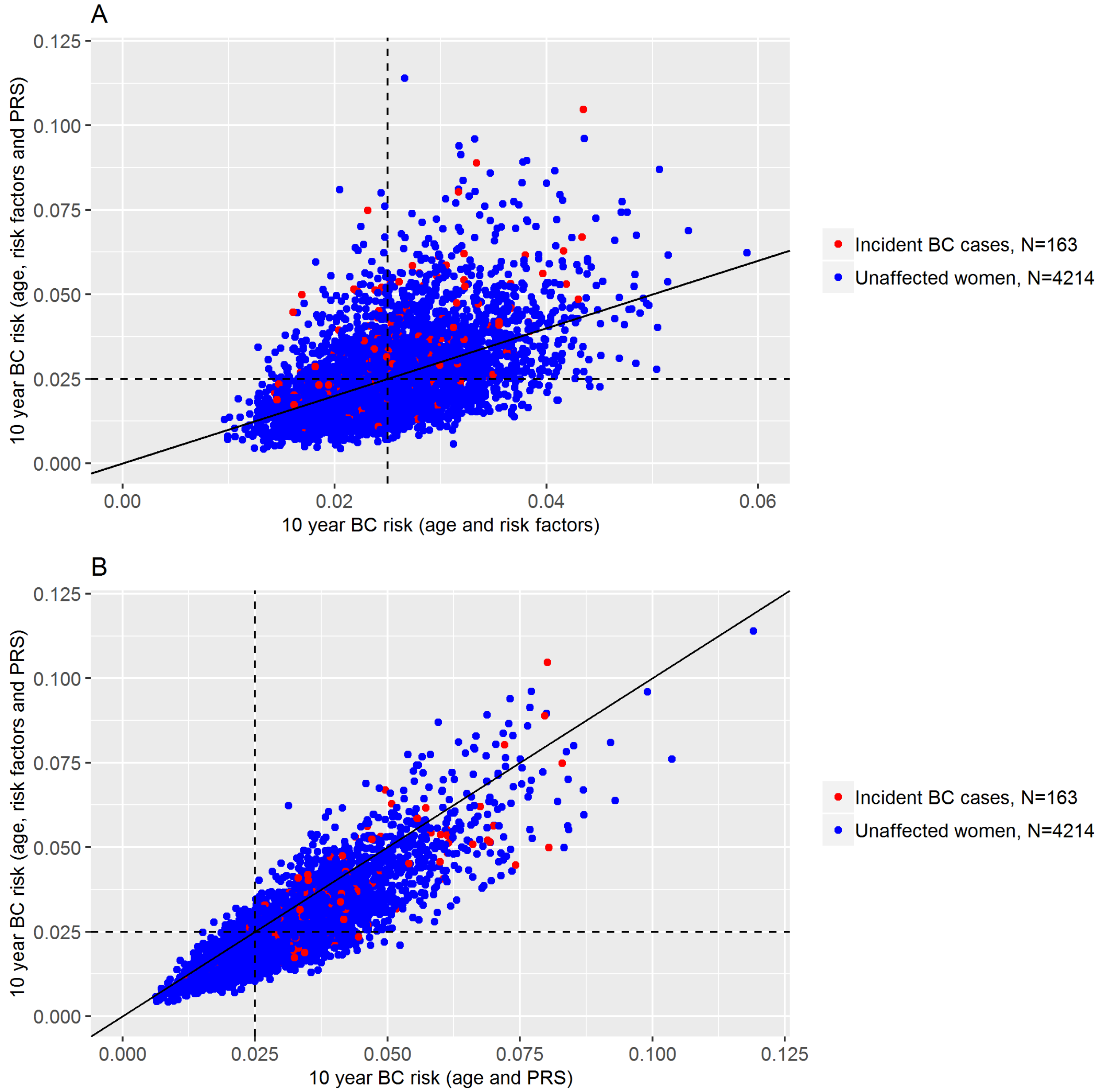

Figure S5: Change in 10-year risk by adding risk factors or the PRS_{313} in the BOADICEA model

Abbreviations: BC, Breast Cancer; BOADICEA, Breast and Ovarian Analysis of Disease Incidence and Carrier Estimation Algorithm; PRS, Polygenic Risk Score.

10-year cumulative BC risks were calculated for all women included in the Rotterdam Study before the age of 70 years, using BOADICEA v5. Women were considered as incident BC cases if they developed BC within 10 years of follow up (shown in red). (A) Risk-change by adding the PRS_{313} in the BOADICEA model (y-axis) including age and risk factors (x-axis). (B) Risk-change by adding risk factors in the BOADICEA model (y-axis) including age and the PRS_{313} (x-axis).

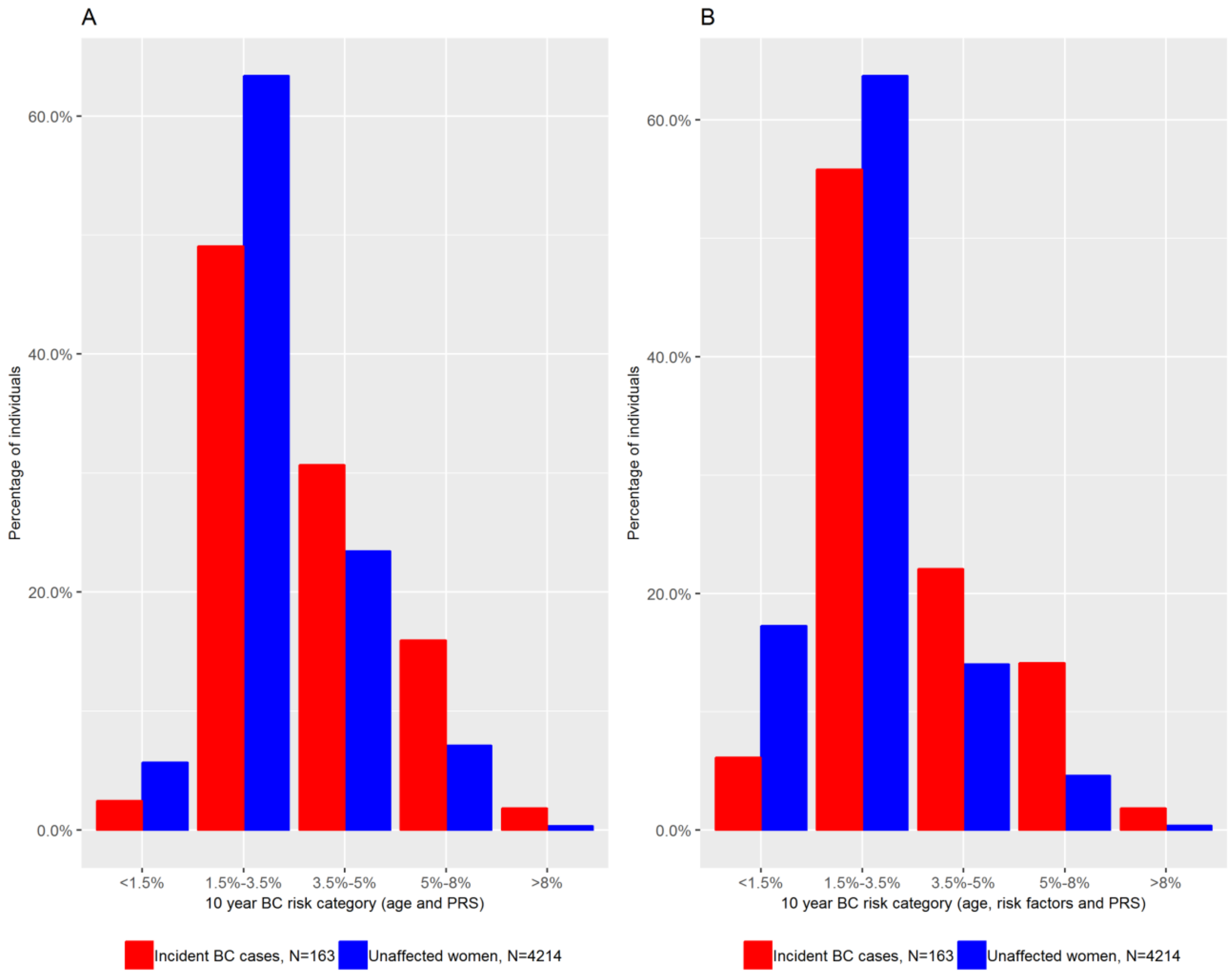

Figure S6: Percentage of unaffected women and incident breast cancer cases in different 10-year risk categories

Abbreviations: BC, Breast Cancer; BOADICEA, Breast and Ovarian Analysis of Disease Incidence and Carrier Estimation Algorithm; PRS, Polygenic Risk Score.

Bar plot of the percentages of women assigned to the different 10-year cumulative BC risk categories (<1.5%; 1.5%-3.5%; 3.5%-5%; 5%-8%; >8%) as calculated with BOADICEA v5 using two sets of variables. Including age and the PRS_{313} (A) and including age, risk factors and the PRS_{313} (B). These risks were calculated for all women included in the Rotterdam Study below the age of 70 years. Women were considered affected if they developed BC within 10 years of follow up.

Supplementary tables

Table S1: Characteristics of the Rotterdam Study cohort

		Total cohort		Subcohort[a]	
		Unaffected	Incident BC	Unaffected	Incident BC
Number		6202	320	4214	163
Rotterdam Study cohort	RS-I	3536	227	1821	152
	RS-II	1057	59	796	50
	RS-III	1609	34	1525	33
Birth cohort	<1900	54	1	0	0
	1900-1910	487	9	0	0
	1910-1920	996	46	0	0
	1920-1930	1441	106	976	77
	1930-1940	1293	97	1235	97
	1940-1950	1087	42	1087	82
	1950-1960	811	18	811	18
	1960	33	1	33	1
Age at inclusion	Mean	66.1	65	59.9	60.4
	Range	45.8-99.2	45.8-96.3	45.8-70.0	45.8-70.0
Age at diagnosis	Mean	-	72.7	-	65.3
	Range	-	48-100	-	48.0-79.0
Invasiveness first BC	Invasive	-	286	-	142
	In situ	-	34	-	21
Asynchronous second BC[b]	All	-	59	-	44
	Invasive	-	59	-	44
	In situ	-	0	-	0
Other incident tumour[c]		728	16	450	13
Risk factors					
Height in cm	Mean	162.3	163.0	164.0	164.3
	Unknown	137 (2%)	5 (2%)	9 (0.2%)	3 (2%)
Alcohol use in grams per day	Mean	6.3	7.1	6.8	6.8
	Unknown	506 (8%)	11 (3%)	742 (18%)	34 (21%)
Age menarche	Mean	13.5	13.3	13.4	13.3
	Unknown	317 (5%)	11 (3%)	102 (2%)	4 (2%)
Age menopause	Mean	48.8	49.2	48.6	49.4
	Unknown	473 (8%)	24 (8%)	255 (6%)	15 (9%)

	Premenopausal	-	-	187	5
Number of children	0	482	25	408	15
	1	811	39	642	22
	2	1819	93	1549	52
	>2	1443	80	1031	41
	Unknown	1647 (27%)	83 (26%)	584 (14%)	33 (20%)
Age at first childbirth	Mean	25.2	25.2	25.0	25.6
	Unknown[d]	47 (1%)	5 (2%)	603 (14%)	34 (21%)
Use of oral contraception	Never	2346	137	1238	50
	Ever	2774	126	2665	90
	Unknown	1082 (17%)	57 (18%)	311 (7%)	23 (14%)
Use of hormone replacement therapy	Never	5050	254	3416	128
	Ever	994	62	758	32
	Unknown	158 (2.5%)	4 (1%)	40 (1%)	3 (2%)
Body Mass Index	Mean	27.0	27.7	27.0	27.8
	Unknown	141 (2%)	5 (2%)	38 (1%)	3 (2%)
Standardised PRS_{313}	Mean	0	0.45	-0.01	0.57
	SD	1.00	1.05	1.00	1.02

Abbreviations: BC, Breast Cancer; PRS, Polygenic Risk Score; RS, Rotterdam Study; SD, Standard Deviation.

[a] Subcohort of women with an age of inclusion in the Rotterdam Study up to age 70

[b] Development of a second primary breast tumour at least one year after the first primary breast tumour.

[c] For women who developed BC during follow up, other tumours were only reported in this study if the other tumour was diagnosed before the BC diagnosis.

[d] For women known to have children.

Table S2: 313 breast cancer associated variants included in the Polygenic Risk Score

First 7 columns of the table are published by Mavaddat et al.[2]

Table S3: Number of included women diagnosed with other type of tumours

ICD10	Tumour description[a]	Unaffected women	Incident BC cases[b]	Total
C00	Lip	5		5
C02	Tongue	2		2
C03	Gum	1		1
C04	Floor of mouth	1		1
C05	Palate	2		2
C06	Mouth	2		2
C08	Major salivary glands	1		1
C09	Tonsil	2		2
C10	Oropharynx	1		1
C15	Oesophagus	27		27
C16	Stomach	21		21
C17	Small intestine	3	1	4
C18	Colon	90	1	91
C19	Rectosigmoid	33	2	35
C20	Rectum	38	1	39
C21	Anus and anal canal	5		5
C22	Liver and intrahepatic bile ducts	8		8
C23	Gallbladder	2		2
C24	Biliary tract	6		6
C25	Pancreas	44		44
C26	Digestive organs	4		4
C32	Larynx	1		1
C34	Bronchus & lung	112		112
C39	Respiratory system and intrathoracic organs	1		1
C40	Bone and articular cartilage of limbs	2		2
C43	Melanoma	27	2	29
C45	Mesothelioma	4		4
C48	Retroperitoneum and peritoneum	1		1
C49	Connective and soft tissue	3		3
C51	Vulva	6		6
C52	Vagina	1		1
C53	Cervix uteri	10	1	11
C54	Corpus uteri	48	2	50

C56	Ovary	24		24
C57	Female genital organs	1		1
C64	Kidney, except renal pelvis	16	1	17
C65	Renal pelvis	4		4
C66	Ureter	1		1
C67	Bladder	24	1	25
C69	Eye and adnexa	5		5
C70	Meninges	1		1
C71	Brain	13		13
C73	Thyroid gland	4	1	5
C80	Malignant neoplasm unspecified	37	1	38
C81	Hodgkin lymphoma	1		1
C82	Follicular lymphoma	6	1	7
C83	Non-follicular lymphoma	13		13
C84	Mature T/NK-cell lymphomas	2		2
C85	Non-Hodgkin lymphoma	11		11
C88	Immunoproliferative diseases	1		1
C90	Multiple myeloma and malignant plasma cell neoplasms	19	1	20
C91	Lymphoid leukaemia	12		12
C92	Myeloid leukaemia	17		17
C93	Monocytic leukaemia	2		2
Total		728	16	744

Abbreviations: BC, Breast Cancer; ICD, International Classification of Diseases and Related Health Problems

[a] ICD10 tumour description[3]

[b] Other tumours are only reported if a women developed this tumour before the BC diagnosis

Table S4: Descriptives for the standardised PRS$_{313}$

		Number	Mean	SD	SE	95% CI
**Unaffected**	**Total**	6202	0.00	1.00	0.01	-0.02-0.02
	**Without other tumour**	5360	-0.01	1.01	0.01	-0.03-0.02
	**Incident other tumour**[a]	728	0.03	0.98	0.04	-0.04-0.10
**Incident BC cases**	**Total**	320	0.45	1.05	0.06	0.34-0.57
	**Invasive BC**	286	0.46	1.05	0.06	0.34-0.58
	***In situ* BC**	34	0.36	1.06	0.18	0.00-0.72
	**One primary breast tumour**	244	0.46	1.00	0.06	0.33-0.59
	**Asynchronous second BC**[b]	59	0.51	1.27	0.17	0.19-0.84

Abbreviations: BC, Breast Cancer; CI, Confidence Interval; PRS, Polygenic Risk Score; SD, Standard Deviation; SE, Standard Error.

[a] Women who developed another type of tumour before inclusion in the Rotterdam Study were excluded (N=114)

[b] Development of a second primary breast tumour at least one year after the first primary breast tumour.

References

1. Ikram MA, Brusselle G, Ghanbari M, et al. Objectives, design and main findings until 2020 from the Rotterdam Study. *European journal of epidemiology.* 2020.
2. Mavaddat N, Michailidou K, Dennis J, et al. Polygenic Risk Scores for Prediction of Breast Cancer and Breast Cancer Subtypes. *American journal of human genetics.* 2019;104(1):21-34.
3. ICD-10 Version:2016. 2016. Available from: https://icd.who.int/browse10/2016/en. Accessed March, 2019.
